# Antioxidant or Apoptosis Inhibitor Supplementation in Culture Media Improves Post-Thaw Recovery of Murine Spermatogonial Stem Cells

**DOI:** 10.3390/antiox10050754

**Published:** 2021-05-10

**Authors:** Sang-Eun Jung, Hui-Jo Oh, Jin-Seop Ahn, Yong-Hee Kim, Bang-Jin Kim, Buom-Yong Ryu

**Affiliations:** 1 Department of Animal Science and Technology, Chung-Ang University, Anseong-si 17546, Gyeonggi-do, Korea; tkddms2428@naver.com (S.-E.J.); gmlwh780128@naver.com (H.-J.O.); ahnjs@cau.ac.kr (J.-S.A.); yhkcau@naver.com (Y.-H.K.); 2 Department of Cancer Biology, Perelman School of Medicine, University of Pennsylvania, Philadelphia, PA 19104, USA; bakim@mail.med.upenn.edu

**Keywords:** permatogonial stem cells, post-thaw recovery, reactive oxygen species, antioxidant, apoptosis

## Abstract

We postulated that supplementation of antioxidant or apoptosis inhibitor in post-thaw culture media of spermatogonial stem cells (SSCs) alleviates reactive oxygen species (ROS) generation and apoptosis. Our aim was to develop an effective culture media for improving post-thaw recovery of SSCs. To determine the efficacy of supplementation with hypotaurine (HTU), α-tocopherol (α-TCP), and Z-DEVD-FMK (ZDF), we assessed the relative proliferation rate and SSC functional activity and performed a ROS generation assay, apoptosis assay, and western blotting for determination of the Bax/Bcl-xL ratio, as well as immunocytochemistry and real-time quantitative polymerase chain reaction (RT-qPCR) for SSC characterization. The relative proliferation rates with HTU 400 μM (133.7 ± 3.2%), α-TCP 400 μM (158.9 ± 3.6%), and ZDF 200 μM (133.1 ± 7.6%) supplementation were higher than that in the DMSO control (100 ± 3.6%). ROS generation was reduced with α-TCP 400 μM (0.8-fold) supplementation in comparison with the control (1.0-fold). Early apoptosis and Bax/Bcl-xL were lower with α-TCP 400 μM (2.4 ± 0.4% and 0.5-fold) and ZDF 200 μM (1.8 ± 0.4% and 0.3-fold) supplementation in comparison with the control (5.3 ± 1.4% and 1.0-fold) with normal characterization and functional activity. Supplementation of post-thaw culture media with α-TCP 400 μM and ZDF 200 μM improved post-thaw recovery of frozen SSCs via protection from ROS generation and apoptosis after cryo-thawing.

## 1. Introduction

Spermatogonial stem cells (SSCs) are the basis for spermatogenesis through self-renewal and differentiation in the outer wall of the seminiferous tubules of testes. Even if the proportion of SSC is only ~0.01% in mice testes, one SSC can theoretically produce 4096 sperm cells that eventually produce progeny for the next generation via fertilization [1]. Therefore, SSCs are a pivotal resource for continuity of the male germline. SSC cryopreservation can facilitate the application of these cells in various fields (e.g., restoration of male fertility in the clinic, preservation of valuable animal breeding stocks, and stem cell research) [2,3,4]. However, cryopreservation is associated with problems such as apoptotic cell death followed by reactive oxygen species (ROS) attack after cryo-thawing, which reduces the efficiency of post-thaw recovery [5,6].

To overcome these ROS attacks in frozen cells, attempts have been made to develop freezing media containing antioxidants and/or apoptosis inhibitors [7,8,9]. In particular, hypotaurine (HTU), a sulfur-containing amino acid with antioxidant capacity, has shown beneficial effects as a cryoprotectant in freezing media for germ cell cryopreservation [9,10,11]. Moreover, α-tocopherol (α-TCP), a non-enzymatic antioxidant, has also shown protective effects against ROS-induced protein damage, lipid peroxidation, and inhibition of cellular mechanisms in germ cell cryopreservation [12,13,14]. Additionally, supplementation of Z-DEVD-FMK (ZDF), a caspase-3 inhibitor, in freezing media has been shown to attenuate cryoinjury-induced apoptosis in mesenchymal stem cell cryopreservation [15].

Despite these efforts, however, these freezing media still have limited ability to reduce cryoinjury of cultured cells after thawing. Furthermore, because major cryoinjury happens in the early stages after cryo-thawing [16], new approaches are required to suppress post-thaw cryoinjury. Accordingly, many reports have demonstrated that pre- or post-cryopreservation care using a culture media with antioxidants or apoptosis inhibitors would allow improved post-thaw recovery in sperm, oocytes, embryos, human embryonic stem cells, and human mesenchymal stem cells [7,17,18,19,20,21,22]. They also demonstrated that supplementation in post-thaw culture media is more effective than that in freezing media. Nevertheless, no previous studies have assessed the efficiency of culture media with antioxidants and apoptosis inhibitors in post-thaw recovery in SSC cryopreservation.

In this regard, we postulated that supplementation of antioxidants or apoptosis inhibitors in post-thaw culture media would alleviate ROS generation and the apoptosis of cryo-thawed SSCs. We aimed to develop an effective culture media for improving post-thaw recovery of SSCs. This study provides a promising approach to improve post-thaw recovery of SSC, which can facilitate widespread SSC utilization in male infertility clinics and/or animal industries.

## 2. Materials and Methods

### 2.1. Experimental Animals

The animal experiments were approved by the Animal Care and Use Committee of Chung-Ang University (Permit Number: 201900048) and were conducted according to the Guide for the Care and Use of Laboratory Animals published by the National Institutes of Health. The animal room was maintained at a constant temperature of 23 ± 1 °C and humidity of 55 ± 10% under a 12-h light/dark cycle. The C57BL/6 (designated C57; DooYeol Biotech, Seoul, Korea) and C57BL/6-TG-EGFP (designated C57-GFP; Jackson Laboratory, Bar Harbor, ME, USA) strains were used as recipient and donor mice, respectively. The donor cells derived from C57-GFP mice could be easily distinguished from those of the C57 recipient mice in in vivo transplantation since the donor cells expressed the enhanced green fluorescent protein (EGFP).

### 2.2. Isolation and Culture of Germ Cells Enriched for SSCs

Unless otherwise noted, all reagents were purchased from Sigma–Aldrich (St. Louis, MO, USA). A total of five cell lines isolated from each litter were used in this study. Germ cells enriched for SSCs were obtained as previously described [23]. Briefly, testes of 6–8-day-old C57-GFP mice were isolated to obtain germ cells enriched for SSCs. After decapsulation of the tunica albuginea, the seminiferous tubules were washed in Dulbecco’s phosphate-buffered saline (DBPS; Life Technologies, Grand Island, NY, USA) and treated in a 2:1 mixture of 0.25% trypsin-EDTA (Invitrogen, Carlsbad, CA, USA) and 7 mg/mL DNase I (Roche, Basel, Switzerland) in DBPS at 37 °C for 5 min to obtain testicular single cells. The digested testicular cells were filtered through a 40-μm cell strainer (BD Biosciences, San Jose, CA, USA), and the filtered testicular cells were then centrifuged at 600× *g* for 7 min at 4 °C. After removing the supernatant, the pallet was resuspended in Dulbecco’s Modified Eagle Medium (DMEM; Life Technologies) containing 10% fetal bovine serum (FBS), 0.1 mM β-mercaptoethanol, 2 mM L-glutamine, 100 µg/mL streptomycin, and 100 U/mL penicillin to 5 × 10^6^ cells/mL. Erythrocytes and cellular debris were removed using a 30% Percoll gradient. Single cells were incubated with anti-Thy-1 antibody microbeads (Miltenyi Biotech, Auburn, CA, USA) in FBS 1% in DPBS for 15 min at 4 °C and enriched via magnetic-activated cell sorting [24]. Thy-1^+^ germ cells enriched for SSCs (designated germ cells enriched for SSCs) were cultured on mitotically inactivated SIM mouse embryo-derived thioguanine- and ouabain-resistant (STO) feeder cells in a defined mouse serum-free medium (mSFM, Table 1) consisting of 10 ng/mL glial cell line-derived neurotrophic factor (GDNF; R&D Systems, Minneapolis, MN, USA), 75 ng/mL GDNF family receptor alpha 1 (GFRα1; R&D Systems), and 1 ng/mL basic fibroblast growth factor (bFGF; Corning, Midland, MI, USA) [25]. These germ cells enriched for SSCs were passaged once weekly.

### 2.3. Cryopreservation

In our previous study, we developed an effective cryoprotectant for SSC freezing, consisting of DMSO 10%, FBS 10%, and trehalose 200 mM in DPBS [26], which was used as the control in the present study. Trehalose was dissolved in DPBS to prepare solution-1 (trehalose 400 mM in DPBS), and, for freezing, cultured germ cells (2 × 10^6^ cells/vial, 8–18 passages) were first suspended in 500 μL of this solution-1 and immediately diluted with the same volume of solution-2 (DMSO 20% and FBS 20% in DPBS) in a dropwise manner. The cell suspensions were transferred to 1.8-mL cryovials (Corning), placed into a Nalgene^®^ freezing container (Nalgene, Rochester, NY, USA) containing isopropyl alcohol, and stored at −80 °C overnight in a deep freezer. The vials were then transferred and stored in liquid nitrogen for at least one month.

### 2.4. Evaluation of the Relative Proliferation Rate after Antioxidant or Apoptosis Inhibitor Treatment

All reagents were prepared before use. To prepare stock solutions, hypotaurine (HTU; Sigma, H1384, powder), α-tocopherol (α-TCP; Sigma, T3251, liquid), and Z-DEVE-FMK (ZDF; TOCRIS, 2166, lyophilized solid) were dissolved in distilled water, ethanol, and DMSO, respectively, and these were diluted 1:1000 with mSFM before use. The concentration of antioxidant/apoptosis inhibitor was defined as 50–400 μM, including the effective concentrations of each reagent (i.e., 200 μM α-TCP, 100 μM HTU, and 100 μM ZDF) [12,27,28]. After thawing at 37 °C for 2.5 min, the germ cell suspension was diluted with 10 mL of a thawing medium (FBS 10% in Minimum Essential Medium-α; Gibco, Grand Island, NY, USA). After washing in mSFM, the same number of thawed germ cells was plated onto a 24-well plate (2 × 10^5^ cells/well). Immediately after thawing, the germ cells were cultured in media (mSFM with 10 ng/mL GDNF, 75 ng/mL GFRα1, and 1 ng/mL bFGF2) containing different concentrations of each antioxidant or apoptosis inhibitor (Table 2) for 12 h in a 5% CO_2_ incubator at 37 °C, based on the findings suggesting that major cryoinjury occurs within the first 12–24 h after cryo-thawing [16]; the cells were then cultured under normal culture conditions without the antioxidant or apoptosis inhibitor for 6.5 days. Subsequently, the germ cells were dissociated with 0.25% trypsin and manually pipetted. After centrifugation at 600× *g* for 7 min at 4 °C, the pellet was resuspended in mSFM, and the relative proliferation rate (%) was calculated as follows:

Proliferation rate (%) = number of cells recovered after cryo-thawing and culture × 100/number of initial cells plated after cryo-thawing (2 × 10^5^) (1)

Relative proliferation rate (%) = proliferation rate of treatment groups × 100/proliferation rate of control groups(2)

The control group included frozen germ cells with no treatment.

### 2.5. ROS Generation Assay

After cryo-thawing, the resuspended germ cells (5 × 10^4^ cells/well) were seeded on wells of a 96-well plate. At 12 h after incubation without or with antioxidants or the apoptosis inhibitor, a DCFDA cellular ROS detection assay (Abcam, Cambridge, UK) was used according to the manufacturer’s instructions to analyze the ROS level within the germ cells enriched for SSCs. Briefly, the germ cells enriched for SSCs were washed by DPBS to eliminate antioxidants and incubated with 25 μM DCFDA solution in a 5% CO_2_ incubator at 37 °C for 30 min. Fluorescence intensity was measured by a fluorescence microplate reader (Gemini XS, Molecular Devices, Sunnyvale, CA, USA) for excitation and emission spectra at 485 and 535 nm, respectively.

### 2.6. Apoptosis Assay

Germ cells were harvested 12 h after thawing and culturing without or with each reagent in a 5% CO_2_ incubator at 37 °C. After washing in cold DPBS, the pellet was resuspended in 1× binding buffer (BD Biosciences), and the cell suspension (1 × 10^5^ cells) was then transferred to a new tube. The cell suspension was incubated in annexin V-APC (BD Biosciences) and propidium iodide (PI) for 15 min at RT (20–25 °C) in the dark. Apoptotic cells were assessed using flow cytometry with a FACSAria II cell sorter (BD Biosciences) equipped with BD CellQuest^TM^ Pro software (Becton Dickinson, Oxford, UK).

### 2.7. Western Blot

Germ cells were collected after 12 h of incubation without or with each antioxidant or apoptosis inhibitor in a 5% CO_2_ incubator at 37 °C. Proteins were extracted using an RIPA buffer (Thermo Fisher Scientific, Rockford, IL, USA) consisting of protease inhibitor and phosphatase inhibitor cocktails (Thermo Fisher Scientific). The lysate was centrifugated at 13,000 rpm for 20 min at 4 °C, after which supernatants were collected. Protein quantification was conducted using a BCA protein assay (Thermo Fisher Scientific), and 5 µg of each protein was loaded onto a 15% SDS-polyacrylamide gel. The separated protein was blotted onto a polyvinylidene difluoride (PVDF) membrane (Millipore, Billerica, MA, USA), which was blocked with 5% skim milk in DPBS containing 0.2% Tween 20 (PBS-T) at RT (20–25 °C) for 2 h. After washing in PBS-T, the membrane was incubated with primary antibodies diluted 1:1000 at 4 °C overnight: rabbit anti-Bcl-xL (2764S, Cell Signaling Technology (CST), Danvers, MA, USA) and rabbit anti-Bax (14792, CST). Mouse anti-α-tubulin (ab7291, Abcam) at a 1:5000 dilution was used as a loading control. After washing in PBS-T, HRP-conjugated secondary antibody diluted 1:2000 was treated at RT (20–25 °C) for 2 h; anti-rabbit IgG (7074S, CST) and anti-mouse IgG (7076S, CST) were used as secondary antibodies. Protein expression was detected using the electrochemiluminescence (ECL) method, and the band intensity was evaluated using ImageJ software (version 1.8.0, National Institutes of Health, Bethesda, MD, USA).

### 2.8. Immunofluorescence

After a week of in vitro culture following cryo-thawing and treatment with the reagents, the germ cells were collected and fixed with 4% paraformaldehyde (Biosesang, Seongnam, Korea) at RT (20–25 °C) for 30 min. The cells were then permeabilized with 0.1% Triton X-100 (*v*/*v*) in DPBS at RT (20–25 °C) for 10 min. After blocking with 5% bovine serum albumin for 1 h, followed by overnight incubation with primary antibodies diluted 1:200 at 4 °C overnight [rabbit anti-promyelocytic leukemia zinc finger (PLZF; NBP1-80894, Novus Biologicals, Centennial, CO, USA), rabbit anti-glial-derived neurotrophic factor family receptor alpha 1 (GFRα1; ab8026, Abcam), rabbit anti-DEAD-box polypeptide 4 (DDX4, also known as VASA; ab13840, Abcam), and mouse anti-KIT proto-oncogene receptor tyrosine kinase (c-Kit; sc-365504, Santa Cruz Biotechnology, Santa Cruz, CA, USA)], the cells were washed three times with DPBS, followed by incubation with Alexa Fluor 568-conjugated anti-rabbit IgG (A11011, Invitrogen) or Alexa Fluor 568-conjugated anti-mouse IgG (A11004, Invitrogen) at RT (20–25 °C) for 1 h. The cellular nuclei were stained using VectaShield^®^ mounting medium containing 4,6-diamidino-2-phenylindole (DAPI, LSBio, Seattle, WA, USA). To analyze the marker expression of GFP^+^ germ cells (%), a TS-1000 microscope interfaced with the NIS Elements imaging software (Nikon, Tokyo, Japan) was used. The number of labeled cells among the GFP^+^ germ cells was calculated in five different microscopic fields by dividing them into five compartments (i.e., upper right/left, lower right/left, and center) to avoid overlap. All spots were visually evaluated, and compartments with less than 50 cells present were excluded.

### 2.9. Real-Time Quantitative Polymerase Chain Reaction (RT-qPCR)

Total RNA was extracted using the TRIzol reagent (Invitrogen) and purified using the PureLink^TM^ RNA Mini Kit (Invitrogen) according to the manufacturer’s recommendations, which was followed by quantification with the NanoDrop spectrophotometer. Total RNA (1500 ng) was used for complementary DNA synthesis with the SuperScript IV First-Strand Synthesis System (Invitrogen) and oligo-(dT) primers. RT-qPCR was performed with cDNA (300 ng) in addition to SYBR Green PCR Master Mix and each gene-specific primer [Ets variant 5 (*Etv5*), LIM homeobox 1 (*Lhx1*), DAZ-like (*Dazl*), and synaptonemal complex protein 1 (*Sycp1*); Glyceraldehyde-3-phosphate dehydrogenase (*Gapdh*); Table 3]. *Gapdh* was used as the internal control. The RT-qPCR assays were performed on a 7500 Real-Time PCR System (Applied Biosystems, Carlsbad, CA, USA) with the following conditions: initial denaturation at 95 °C for 10 min, followed by 40 cycles of 95 °C for 15 s and 60 °C for 1 min in a two-step thermal cycle, and a final melting curve step at 95 °C for 15 s, 60 °C for 1 min, 95 °C for 30 s, and 60 °C for 15 s. All quantification cycle (C_T_) values were normalized to the *Gapdh* levels, and quantification was performed using the 2^−ΔΔCT^ method.

### 2.10. In Vivo Transplantation

The recipient 6-week-old C57 mice were prepared by intraperitoneal injection of 45 mg/kg body weight of busulfan to exclude endogenous germ cells. Early-passage (8–11 passages) GFP^+^ germ cells were used as donor cells and concentrated to a density of 1.0 × 10^6^ cells/mL for transplantation. Ketamine (75 mg/kg) and medetomidine (0.5 mg/kg) were used to anesthetize the C57 recipient mice. The C57-GFP^+^ donor cells were transplanted into the recipient mice testes through efferent ducts, as previously described [29]. Two months after transplantation, the recipient mouse testes were collected, and the tunica albuginea were decapsulated to analyze colony formation. To quantify the SSCs, the number of C57-GFP^+^ donor colonies greater than 1 mm in length was counted via fluorescence microscopy [30]. To determine whether the stemness of the SSCs was maintained, the number of colonies per 10^5^ cells transplanted, which is also known as the ratio of SSCs to 10^5^ cells transplanted, was calculated as follows:

Colonies/10^5^ cells transplanted = (number of colonies × 10^5^)/number of transplanted cells (3)

To demonstrate the efficiency of post-thaw recovery on the SSCs, the total number of SSCs within the recovered germ cells after freezing, thawing, and culturing was quantified as follows:

Colonies/total number of cultured SSCs after cryopreservation = (number of colonies × total number of cultured cells)/number of transplanted cells(4)

### 2.11. Statistical Analysis

All statistical analyses were conducted with SPSS version 20 (IBM; Armonk, NY, USA). The Shapiro–Wilk and Levene’s tests were used to assess normal distribution and homogeneity of variance, respectively, and multiple comparisons between samples were conducted using one-way analysis of variance (ANOVA) coupled with the post-hoc Tukey’s honestly significant difference test. Dunnett’s test was used to determine whether each of the treatments showed a significant difference from the control for screening of dose-dependent effects. All experiments were conducted at least in triplicate unless otherwise stated, and all data were reported as the mean ± SEM. A significance level of *p* < 0.05 was deemed statistically significant.

## 3. Results

### 3.1. Supplementation of Antioxidants or Apoptosis Inhibitors in the Post-Thaw Culture Media Improves the Proliferation Rates of Germ Cells

**Figure 1:**
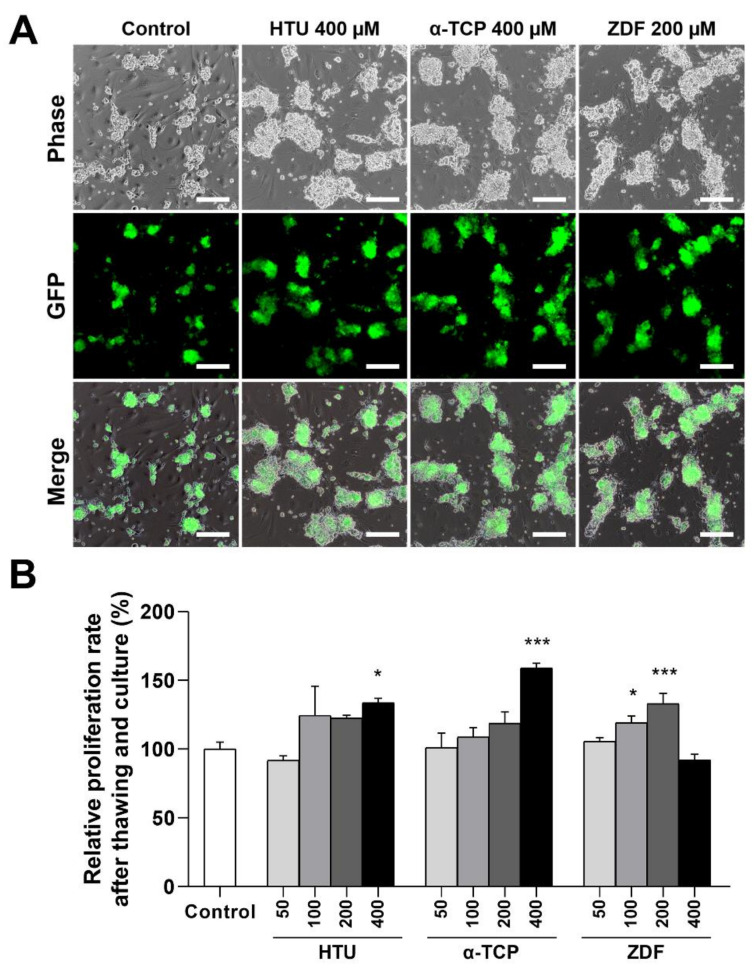
Effects of post-thaw recovery with antioxidant or apoptosis inhibitor supplementation on frozen germ cells. (**A**) Bright/dark-field images of GFP^+^ germ cells after post-thaw recovery for 12 h followed by in vitro culture for one week. Scale bars = 100 μm (10×). (**B**) Relative proliferation rate after post-thaw recovery with antioxidant or apoptosis inhibitor supplementation at different concentrations. The cells in the control group received no treatment after cryo-thawing. The values are expressed as the mean ± SEM (*n* = 5). Statistical analyses were performed using one-way ANOVA coupled with Dunnett’s post-hoc test. Asterisks indicate statistical significance at *p* < 0.05 (*) and *p* < 0.001 (***). HTU, Hypotaurine; α-TCP, α-tocopherol; ZDF, Z-DEVD-FMK.

The relative proliferation rate was evaluated to assess the efficiency of antioxidant or apoptosis inhibitor supplementation in increasing post-thaw recovery. With normal-shaped germ cell clumps (Figure 1A), the relative proliferation rate was significantly higher in the groups supplemented with HTU 400 μM (133.7 ± 3.2%), α-TCP 400 μM (158.9 ± 3.6%), and ZDF 100 and 200 μM (119.3 ± 3.2% and 133.1 ± 7.6%, respectively) in comparison with the controls (100 ± 3.6%) in which the cells received no treatment after cryo-thawing (Figure 1B). The cells in the control group received no treatment after cryo-thawing. Therefore, we chose HTU 400 μM, TCP 400 μM, and ZDF 200 μM as the optimal concentrations for assessing the benefits for post-thaw recovery and used these concentrations in all downstream experiments.

### 3.2. Protective Effects of Antioxidants and the Apoptosis Inhibitor in the Post-Thaw Recovery Phase

**Figure 2:**
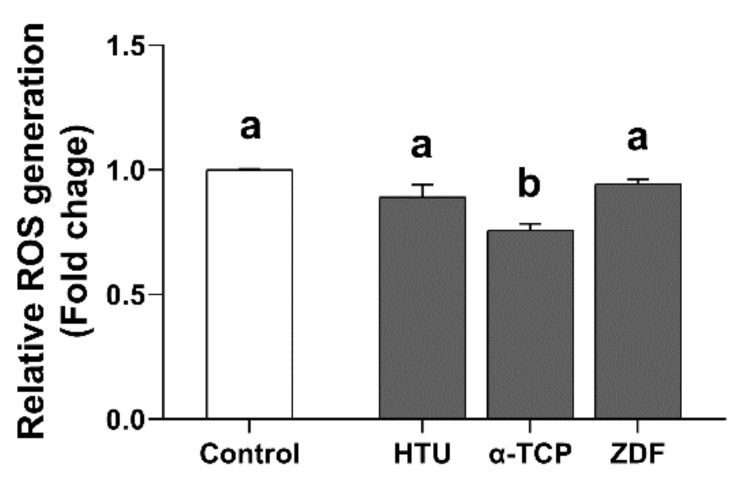
ROS generation in frozen germ cells after post-thaw recovery with antioxidant or apoptosis inhibitor supplementation. The ROS level was evaluated 12 h after post-thaw recovery in cryo-thawed germ cells. The values are expressed as the mean ± SEM (*n* = 3). Statistical analysis was performed using one-way ANOVA coupled with Tukey’s post-hoc test. Different letters above each column indicate statistically significant differences (*p* < 0.05). HTU, Hypotaurine; α-TCP, α-tocopherol; ZDF, Z-DEVD-FMK.

**Figure 3:**
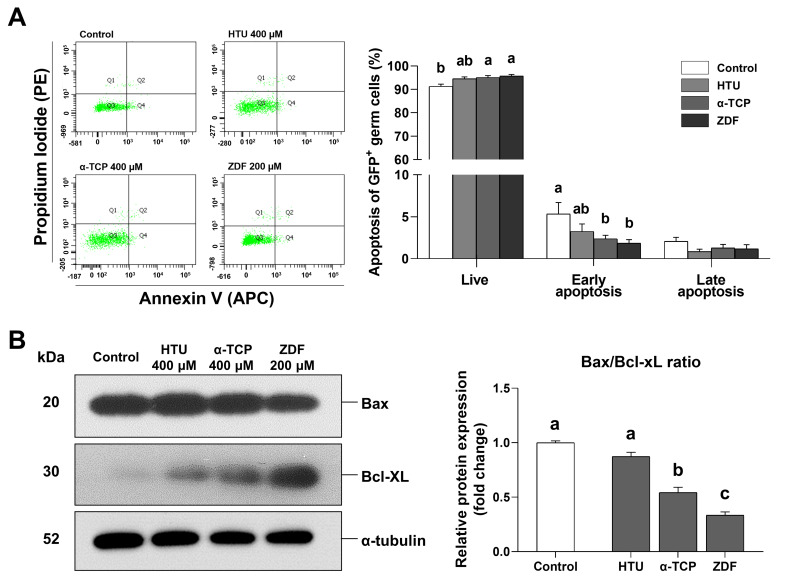
Apoptosis of frozen germ cells after post-thaw recovery with antioxidant or apoptosis inhibitor supplementation. (**A**) Early and late apoptosis in the HTU 400 μM, α-TCP 400 μM, and ZDF 200 μM treatment groups. Apoptosis was evaluated 12 h after post-thaw recovery in cryo-thawed germ cells. The apoptotic cell population is summarized in a graph. (**B**) Bax/Bcl-xL ratio determined using western blot analysis. α-Tubulin was used as a loading control. The graph represents the quantification of protein expression, which was measured as the fold change of the treatment groups with respect to the control. The values are expressed as mean ± SEM (*n* = 3). Statistical analysis was performed using one-way ANOVA coupled with Tukey’s post-hoc test. Different letters above each column indicate statistically significant differences (*p* < 0.05). HTU, Hypotaurine; α-TCP, α-tocopherol; ZDF, Z-DEVD-FMK.

The ROS generation and apoptosis levels were assessed to identify the causes/mechanisms underlying the improved post-thaw recovery of frozen germ cells. Our results showed that the ROS generation level was significantly reduced by α-TCP 400 μM (0.8 ± 0.0-fold) treatment in comparison with the control group (1.0 ± 0.0-fold), whereas no significant difference was observed between the control group (1.0 ± 0.0-fold) and the HTU 400 μM (0.9 ± 0.0-fold) and ZDF 200 μM (0.9 ± 0.0-fold) groups (Figure 2). Although apoptosis level data showed no significant difference in late apoptosis, early apoptosis was significantly lower in the groups supplemented with α-TCP 400 μM (2.4 ± 0.4%) and ZDF 200 μM (1.8 ± 0.4%) in comparison with the control (5.3 ± 1.4%; Figure 3A). Additionally, the relative expression ratio of Bax/Bcl-xL was also significantly decreased by α-TCP 400 μM (0.5 ± 0.0-fold) and ZDF 200 μM (0.3 ± 0.0-fold) in comparison with the control (1.0 ± 0.0-fold; Figure 3B). Thus, our results indicate that α-TCP 400 μM and ZDF 200 μM treatments in the post-thaw recovery phase effectively suppressed ROS generation or the apoptosis level in frozen germ cells after thawing.

### 3.3. Stable Characterization after Post-Thaw Recovery with Antioxidants or Apoptosis Inhibitors

**Figure 4:**
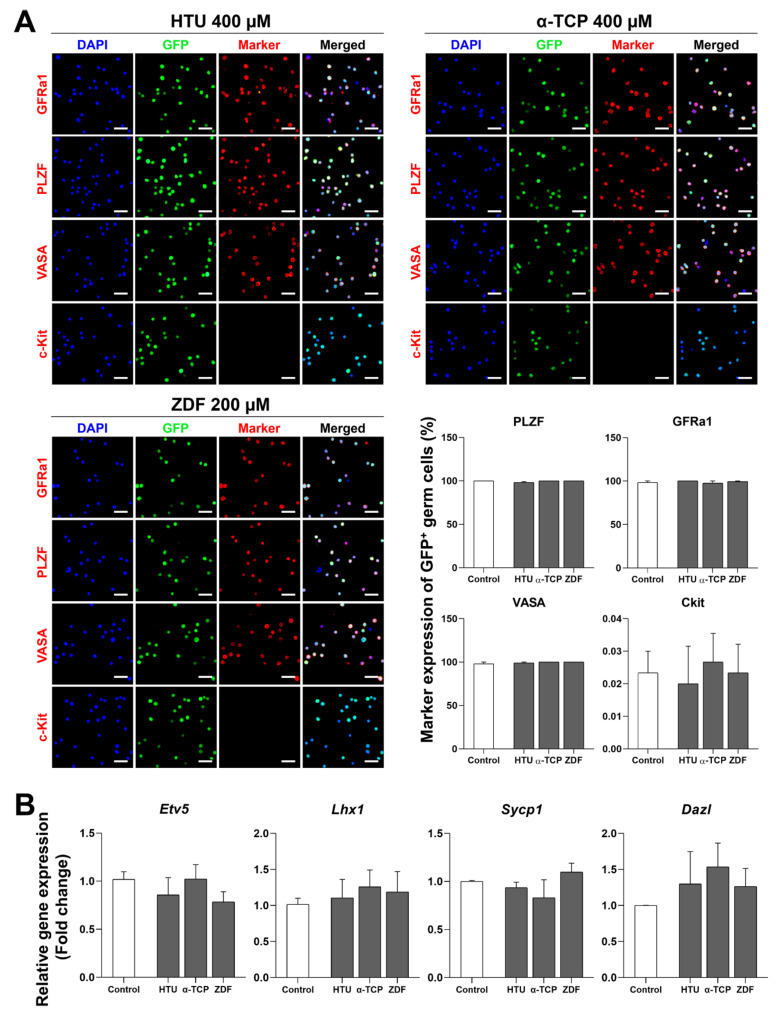
Characterization of germ cells after post-thaw recovery with antioxidant or apoptosis inhibitor supplementation. (**A**) Immunofluorescence analysis of GFP^+^ germ cells after post-thaw recovery in the HTU 400 μM, α-TCP 400 μM, and ZDF 200 μM treatment groups. Representative images obtained after immunofluorescence analyses using markers (shown in red) for undifferentiated spermatogonia (GFRα1 and PLZF), germ cells (VASA), and differentiated spermatogonia (c-Kit). Nuclei are labeled with DAPI (blue). Scale bar = 100 µm (40×). Marker expression in GFP^+^ GSCs is summarized as a bar graph. (**B**) Relative gene levels of germ cells after post-thaw recovery. Genes for undifferentiated spermatogonia (*Etv5* and *Lhx1*) and differentiated spermatogonia (*Sycp1* and *Dazl*) are shown in relative graphical data. Values are expressed as the mean ± SEM (*n* = 3). Statistical analysis was performed by one-way ANOVA with Tukey’s test as a post-hoc test. HTU, Hypotaurine; α-TCP, α-tocopherol; ZDF, Z-DEVD-FMK.

Unlike other cells, safety assessment is especially essential for germ cell freezing because germ cells are supposed to transfer their genetic information to subsequent generations. Therefore, germ cells were characterized using immunofluorescence and RT-qPCR after post-thaw recovery with HTU 400 μM, α-TCP 400 μM, or ZDF 200 μM supplementation. Our immunofluorescence data showed normal expression of GFRα1 and PLZF (markers for undifferentiated spermatogonia) as well as VASA (a marker for germ cell lineage), with no significant difference between the control and treatment groups (Figure 4A). Likewise, the expression of c-Kit (a marker for differentiated spermatogonia) was within the normal range and showed no significant differences in all treatment groups. Similarly, RT-qPCR analyses showed no significant differences in the expression of *Etv5* and *Lhx1* (markers for undifferentiated spermatogonia), as well as *Dazl* and *Sycp1* (markers for differentiated speramtogonia) among the groups (Figure 4B). Thus, our findings indicate that the normal characteristics of germ cells were maintained after post-thaw recovery with HTU 400 μM, α-TCP 400 μM, or ZDF 200 μM supplementation.

### 3.4. Functional Activity of SSCs after Post-Thaw Recovery with Antioxidant or Apoptosis Inhibitor Supplementation

**Figure 5:**
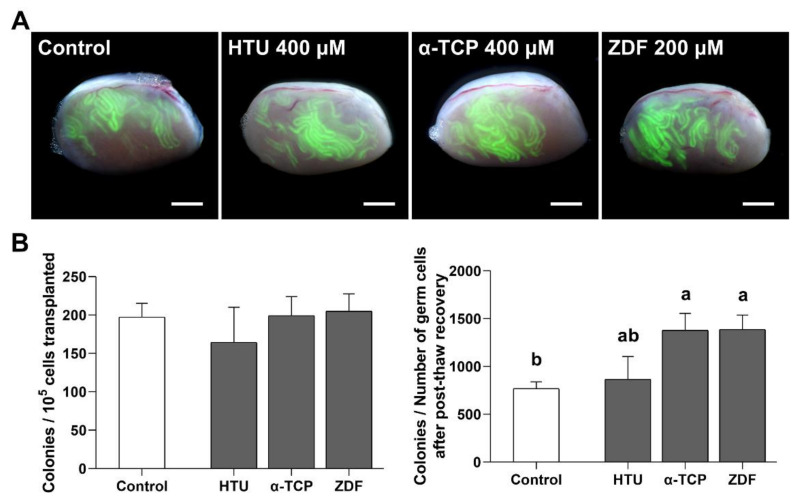
Functional activity of SSCs after post-thaw recovery with antioxidant or apoptosis inhibitor supplementation. (**A**) Merged bright/dark-field fluorescence images of C57 recipient testes after in vivo transplantation. Colonies derived from the donor C57-GFP^+^ SSCs are formed in seminiferous tubules, which are distinguished in the C57 recipient mice. Scale bar = 2 mm. (**B**) Number of colonies per 10^5^ cells transplanted and number of colonies per recovered germ cells after post-thaw recovery and culture. Total number of mice/testes used for analysis was 8/13, 8/11, 8/15, and 8/13 in the control, HTU 400 μM, α-TCP 400 μM, and ZDF 200 μM groups, respectively. Non-injected testes with a poor condition were excluded from testis analysis. Values are expressed as mean ± SEM (*n* = 13, 11, 15, and 13 testes, respectively). A statistically significant difference (*p* < 0.05) is represented by different letters above each column.

Spermatogonial transplantation is unequivocally a crucial tool for investigating the functional activity of SSCs after post-thaw recovery with HTU 400 μM, α-TCP 400 μM, or ZDF 200 μM supplementation. With normal colony formation of SSCs in all groups (Figure 5A), no significant differences were observed in the number of colonies per 10^5^ transplanted germ cells among the control (197.3 ± 18.0 colonies), HTU 400 μM (164.4 ± 45.6 colonies), α-TCP 400 μM (199.3 ± 24.8 colonies), and ZDF 200 μM (205.0 ± 22.5 colonies) groups (Figure 5B). Therefore, we concluded that the population of SSCs in 10^5^ germ cells was constant regardless of the treatment itself as well as the type of treatment (i.e., antioxidant or apoptosis inhibitor). However, when considering the total number of SSCs collected after post-thaw recovery with HTU 400 μM, α-TCP 400 μM, or ZDF 200 μM supplementation, this result could be interpreted differently. The population of SSCs in the total number of germ cells collected after post-thaw recovery showed a significant difference in the α-TCP 400 μM (1378.9 ± 177.2 colonies) and ZDF 200 μM (1387.3 ± 150.3 colonies) groups in comparison with the control (769.5 ± 70.1 colonies) group (Figure 5B). However, there was no difference between the HTU 400 μM (865.4 ± 238.8 colonies) and control (769.5 ± 70.1 colonies) groups. Collectively, the findings indicate that post-thaw recovery with α-TCP 400 μM or ZDF 200 μM supplementation effectively increased the collection of many SSC populations after cryo-thawing.

## 4. Discussion

The development of cryoprotectants has resulted in a gradual increase in the success rates of SSC cryopreservation procedures. Nevertheless, poor post-thaw care of frozen cells can reduce the efficiency of recovery from ROS attacks after thawing. Therefore, we postulated that the post-thaw culture media with an antioxidant or apoptosis inhibitor would enhance post-thaw recovery by alleviating ROS generation and apoptosis. In this study, we aimed to develop post-thaw culture media to improve the recovery of cryo-thawed SSCs.

According to Murray KA et al., evaluation of total cellular recovery via post-thaw culture is crucial to assess the protective effect of a reagent in cryopreservation studies [31]. Therefore, we evaluated the proliferation rate after cryo-thawing and culturing to investigate the efficiency of supplementation with antioxidant or apoptosis inhibitors. Our results indicated that the highest relative proliferation rate of SSCs was observed with supplementation of HTU 400 μM, α-TCP 400 μM, or ZDF 200 μM in the post-thaw recovery phase, suggesting that cryoinjury can be alleviated by using culture media specifically designed for the care of cryo-thawed cells in addition to using an appropriate freezing medium. This may be attributed to the fact that major cryoinjuries occur in the early stage after cryo-thawing rather than during freezing [16], which is supported by a previous study reporting that exposure to apoptosis inhibitors during culture can enhance post-thaw recovery of human stem cells more than its exposure in freezing media [7,22].

HTU is present in most mammalian tissue [32], and HTU-taurine metabolism shows protective effects on the male reproductive system as an excellent scavenger of ROS in vivo [33]. Moreover, HTU positively affects sperm motility, DNA integrity, and lipid peroxidation in humans [34]. Despite these important actions of HTU-taurine, our results showed that the exposure to HTU 400 μM had no effects on ROS generation and apoptosis even though it improved the proliferation rate of cryo-thawed SSCs. This result is likely attributed to difference response to HTU between SSCs and spermatozoa because spermatogonia (including SSCs) are much more tolerant to ROS attack than spermatozoa [35]. Furthermore, since antioxidants are involved in an enormous amount of cellular biochemical process [36], HTU presumably affected SSC proliferation via an unknown mechanism rather than alleviating ROS generation and apoptosis. However, when considering transplantation data, there was no positive efficacy of hypotaurine on the recovery of true SSCs.

In our study, exposure to α-TCP 400 μM during in vitro culture enhanced the efficiency of post-thaw recovery, which is likely due to the role of α-TCP as an antioxidant in protecting the unsaturated lipids in the cellular membrane from ROS-induced damage [37]. This is supported by our results in which ROS generation, early apoptotic cell population, and the Bax/Bcl-xL ratio were considerably reduced by post-thaw exposure of cryo-thawed SSCs to α-TCP. Likewise, in many studies, α-TCP has been shown to suppress cryoinjury of SSCs or sperm via its cryoprotective properties of scavenging free radicals and to prevent lipid peroxidation and/or apoptosis from cryoinjury even when α-TCP was used in freezing media [12,13,14]. Therefore, this study is the first to identify that α-TCP supplementation in post-thaw culture media strongly improves the post-thaw recovery of SSCs.

Additionally, our data showed that exposure to ZDF 200 μM significantly alleviates early apoptosis and reduces the Bax/Bcl-xL ratio, indicating that ZDF can be used as an efficient culture media supplement after thawing in SSC cryopreservation. Although ZDF, a caspase-3 inhibitor, did not directly modulate ROS generation, it may have helped in indirectly suppressing cryoinjury-induced apoptosis because ROS generation is responsible for caspase-3 activation in frozen cells [21,38]. Interestingly, our data indicated that high doses of ZDF considerably reduced its efficiency, thus highlighting the importance of an apoptosis baseline. Previous studies have also reported that, at low to modest levels, apoptosis is essential for regulation of the normal physiological functions involved in the development and survival of multicellular organisms [39,40]. Therefore, we demonstrate that modest prevention of caspase activation by the caspase-3 inhibitor ZDF improves the post-thaw recovery of frozen SSCs.

In procedures utilizing SSCs to form sperm, quality assurance of SSCs is ultimately very important because of the ability of SSCs to transfer male genetic information to subsequent generations throughout spermatogenesis. In this study, the normal characterization and stemness of the cultured SSCs after cryo-thawing post-supplementation with HTU 400 μM, α-TCP 400 μM, or ZDF 200 μM indicated that SSCs maintained their normal properties without spontaneous differentiation. However, in vivo functional activity data showed that the total number of true SSCs in the collected cells was higher in only the α-TCP 400 μM and ZDF 200 μM groups in comparison with the control group. We suggest that supplementation of α-TCP 400 μM or ZDF 200 μM for post-thaw recovery did not negatively affect the functionality of SSCs. Unexpectedly, the total number of true SSCs was decreased in the HTU 400 μM group, which is consistent with the above data showing that HTU 400 μM did not perfectly protect cells from both ROS generation and apoptosis. Thus, we concluded that α-TCP 400 μM and ZDF 200 μM could serve as effective additives to improve post-thaw recovery in SSC cryopreservation through protection of frozen SSCs from ROS generation and apoptosis after cryo-thawing. Additionally, further study is required to determine the beneficial effects of long-term treatment of these additives on ROS alleviation of cryo-thawed SSCs.

## 5. Conclusions

Our study demonstrated that modulation of ROS generation and apoptosis using a culture system with antioxidants or apoptosis inhibitors has a protective effect on frozen SSCs after thawing. Therefore, α-TCP and ZDF improve the pro-survival response of cryo-thawed SSCs by reducing ROS generation and/or apoptosis, facilitating their post-thaw recovery. Taken together, our findings provide a new approach to improve SSC recovery after thawing. Thus, this protocol could facilitate more widespread use of SSCs in the livestock industry and/or male infertility clinics.

## Figures and Tables

**Table 1:** Composition of mouse serum-free media (mSFM)**.**

Ingredients ^a^	Final Concentration
Penicillin	50 unit/mL
Streptomycin	50 μg/mL
Bovine serum albumin	0.2%
Iron-saturated transferrin	10 μg/mL
Free fatty acids	7.8 μEq/L
Na2SeO3	3 × 10^−8^ M
L-Glutamine	2 mM
2-Mercaptoethanol	50 μg
Insulin	5 μg/mL
*N*-2-hydroxyethylpiperazine-*N*′-2-ethanesulfonic acid (HEPES)	10 mM
Putrescine	60 μM

^a^ All ingredients were diluted in Eagle’s minimum essential medium-alpha (MEM-α, Invitrogen).

**Table 2:** Antioxidants and apoptosis inhibitor used for post-thaw recovery.

Reagent	Property	Final Concentration (µM)			
Hypotaurine (HTU)	Antioxidant	50	100	200	400
α-Tocopherol (α-TCP)	Antioxidant	50	100	200	400
Z-DEVD-FMK (ZDF)	Apoptosis inhibitor	50	100	200	400

**Table 3:** RT-qPCR primers.

Gene	Forward Primer (5′→3′)	Reverse Primer (5′→3′)	Accession Number
*Gapdh*	TGACCCCTTCATTGACCTTC	TACTCAGCACCAGCATCACC	NM_008084.3
*Etv5*	CCCGGATGCACTCTTCTCTATG	TCGGATTCTGCCTTCAGGAA	NM_023794
*Lhx1*	CCCAGCTTTCCCGAATCCT	GCGGGACGTAAATAAATAAAATGG	NM_008498
*Scyp1*	CGCTACAACCACATGCTTCG	GGAACGCTGCTTAGATCTCCTC	NM_011516
*Dazl*	AATGTTCAGTTCATGATGCTGCTC	TGTATGCTTCGGTCCACAGACT	NM_010021

## Data Availability

Data is contained within the article.
